# Multi-disciplinary community-based group intervention for fibromyalgia: a pilot randomized controlled trial

**DOI:** 10.1007/s00296-023-05403-5

**Published:** 2023-08-11

**Authors:** Kara Turcotte, Nelly D. Oelke, Gina Whitaker, Susan Holtzman, Brian O’Connor, Neil Pearson, Michelle Teo

**Affiliations:** 1 Department of Nursing, Western University, London, ON Canada; 2 Faculty of Health and Social Development, School of Nursing, The University of British Columbia, 3333 University Way, Kelowna, BC V1V 1V7 Canada; 3 Department of Psychology, University of British Columbia, Kelowna, BC Canada; 4 Faculty of Medicine, School of Physical Therapy, University of British Columbia, Kelowna, BC Canada; 5 Department of Medicine, Faculty of Medicine, University of British Columbia, Kelowna, BC Canada

**Keywords:** Fibromyalgia, Chronic pain, Multidisciplinary intervention, Community-based, Quality of care, Randomized control trial

## Abstract

Fibromyalgia is characterized by widespread pain, fatigue, sleep disturbances, mood disturbances, and cognitive impairment. Most individuals with fibromyalgia experience poorly managed symptoms and increased healthcare service use. Multicomponent therapies, with a focus on nonpharmacological modalities, are increasingly supported in the literature. However, given the limited resources available, implementation in smaller communities remains a challenge. This research tested a community-based multidisciplinary group intervention for individuals diagnosed with FM living in a small urban centre. The primary outcome was perceptions of quality of care and secondary outcomes included disease-related functioning, anxious and depressive symptoms, pain beliefs, and health service utilization. A pilot randomized control trial was conducted in which 60 patients diagnosed with fibromyalgia were randomized into a 10-week community-based multidisciplinary group intervention program or usual care. Treatment components included twice-weekly exercise sessions and weekly education sessions (e.g., pain education, cognitive behavioral strategies for stress, nutrition, peer support). The trial (NCT03270449) was registered September 1 2017. Statistically significant post-intervention improvements were found in the primary outcome, perceived quality of care (Cohen’s *d* = 0.61, 0.66 for follow up care and goal setting, respectively). Secondary outcomes showing statistically significant improvements were disease-related daily functioning (Cohen’s *d* = 0.70), depressive symptoms (Cohen’s *d* = 0.87), and pain beliefs (Cohen’s *d* = 0.61, 0.67, 0.82 for harm, disability and control, respectively). No adverse events were reported. Community-based multidisciplinary group interventions for fibromyalgia show promise for improving satisfaction with quality of care, disease-related functioning, and depression, and fostering more adaptive pain beliefs.

## Introduction

Fibromyalgia (FM) is a heterogenous chronic disease of unknown etiology which affects approximately 2–4% of the general population in North America [1]. FM is characterized by heightened pain sensitivity with symptoms including chronic widespread pain, fatigue, sleep disturbances, mood disturbances, and cognitive impairment [2]. The diagnosis of FM can be challenging and is often preceded by frequent visits to clinicians, multiple diagnostic tests, and long wait-times [3]. The delay in diagnosis may result in delayed treatment and suboptimal care [4]. Patients with FM are often treated with a mostly pharmacological approach, which is known to result in a clinically meaningful improvement in less than half of patients [5]. As a consequence, many individuals are left with unmanaged symptoms. Current treatment approaches for FM have led to fragmented care (i.e., patients perceive a lack of coordination, poor continuity of care, and limited support from healthcare workers), which further contribute to ineffective symptom relief [4, 6–8].

Broadly speaking, instilling strong self-management skills is considered the gold standard approach to sustainable symptom reduction and improved function for chronic pain syndromes, including FM [9]. Current evidence-based guidelines suggest that multicomponent therapies which incorporate nonpharmacological treatment strategies in addition to pharmacological approaches should be a first line of treatment for people with FM [10, 11]. These guidelines, in addition to recent systematic reviews of the research literature [5, 12], have highlighted that integrated and multidisciplinary therapeutic approaches show promise in the management of FM.

Building on emerging findings [12–15], a feasibility study was conducted to evaluate a comprehensive, integrated, community-based model of care for FM [16]. A key outcome demonstrated in this feasibility study was improved patient-perceived quality of care [16]. This is important because when patients have a positive and healthy attitude towards making lifestyle changes, they may be better able to engage in self-management of symptoms, and have reductions in health care utilization.

The objective of the current pilot randomized control trial was to examine the effectiveness of a multidisciplinary, community-based intervention for people with FM living in a small urban centre. The intervention incorporated feedback from the patients and health care providers who participated in the original feasibility trial [16]. Focused research on smaller communities is critical as these communities typically have fewer resources and increased barriers for accessing healthcare [17]. The community setting may offer a more sustainable approach to improving FM care, disease management, and health service use in both rural and urban contexts. Participation in community-based programs, including exercise programs, has been recommended as it is both accessible to most individuals and affordable [1, 18]. The primary outcome in the current study was patient perceptions of overall quality of care, and the secondary outcomes were chosen to assess the impact of the intervention on key symptoms and functional outcomes, including daily functioning, anxious and depressive symptoms, pain beliefs, and health service utilization. It was hypothesized that the intervention would lead to significant improvements post-intervention and at the three-month follow-up in patient-perceived quality of care, daily functioning, anxious and depressive symptoms, as well as significant increases in adaptive pain beliefs, and decreases in healthcare service utilization, compared to usual care.

## Materials and methods

### Design

This pilot study used a non-blinded randomized control trial design with two parallel groups comparing a community-based multidisciplinary group intervention to usual care for individuals diagnosed with FM. A pilot was deemed an appropriate approach given the multi-component nature of the intervention, and that it included modifications made to our original intervention following a feasibility trial [16]. Participants were randomized into either the intervention group or control group using block randomization with a block size of four using Random Allocation Software [19]. Data collection took place over two years, between September 2017 and June 2019. The study protocol was a registered clinical trial (NCT03270449), received institutional ethics board approval on September 15 2017 (H17-01782-A010), and informed consent was obtained from all participants at enrollment.

### Recruitment and participants

The feasibility study [16] demonstrated a large effect size (r = 0.63) on the primary outcome (i.e., Patient Assessment of Chronic Illness Care (PACIC) survey) [20]. Based on this effect size, a power analysis using G*power software indicated that a sample of 60 participants, with 30 participants per group, would be sufficient to achieve 80% power with an alpha of 0.05 [21].

Participants were recruited through local general practitioner and rheumatologists’ offices, as well as through poster advertisements at medical clinics, a local hospital, local FM Facebook group, and other public online and community venues. Study inclusion criteria were: (1) formal diagnosis of FM by a physician based on their clinical assessment (and further confirmed by the study team rheumatologist); (2) resident of the small urban centre (population approximately 35,000) in British Columbia, Canada or surrounding area; (3) at least 19 years of age; (4) fluent in English or able to provide a translator; and (5) capacity to provide informed consent. The study exclusion criteria were: (1) individuals with a severe and/or chronic medical or psychiatric condition that could interfere with their ability to participate in the intervention; (2) women who were pregnant or lactating; (3) patients that did not provide permission to contact their primary health care provider; and (4) individuals who had previously participated in the feasibility study.

**Fig. 1:**
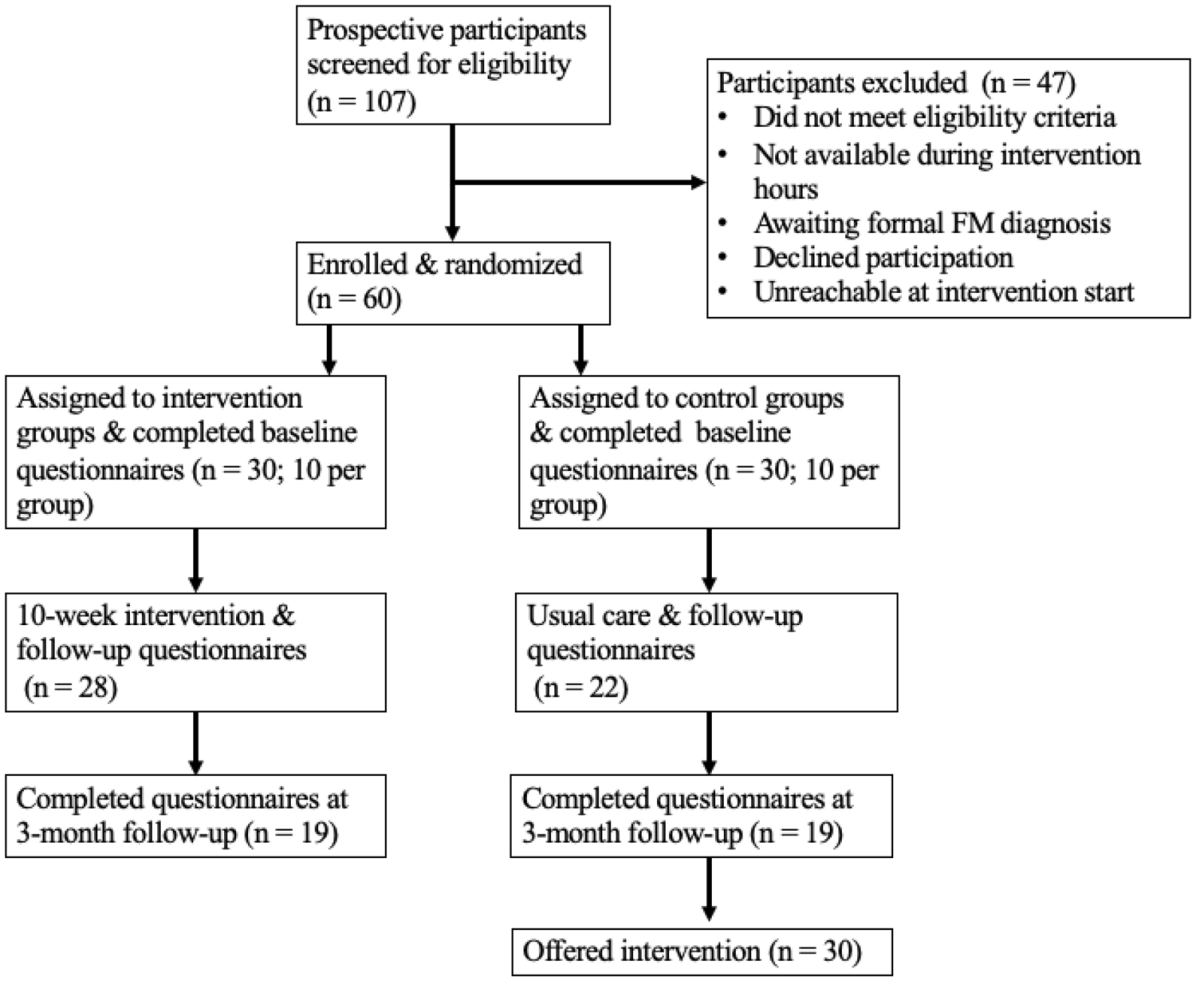
Participant flow through the study

Of the 107 prospective participants screened for eligibility by the study coordinator, 60 were enrolled and randomized to either the intervention (n = 30) or control group (n = 30). Prospective participants who were not randomized (n = 47) either did not meet the inclusion criteria, were not available during the intervention hours, were waiting for formal FM diagnoses, declined to participate, or could not be contacted prior to the intervention start date. See Fig. 1 for a CONSORT flow diagram.

### Intervention group

The treatment was a community-based multidisciplinary group intervention which took place in a small urban centre in British Columbia, Canada. The intervention tested in the current study is a modified version of the original community-based intervention that was tested in an initial feasibility study [16]. As part of this feasibility study, qualitative feedback about the intervention was obtained from 11 patients and 6 health care providers; overall, feedback was positive and participants reported it was a helpful and valuable intervention. Based on participant feedback, several changes were made to the intervention (e.g., participants and health care providers both suggested adding a mental health counsellor component and as such four sessions on cognitive-behavioral therapy strategies for improving mental health were added to the intervention).

The intervention groups were made up of ten individuals per cohort for a total of three cohorts. Each cohort participated in a 10-week intervention which included once-weekly education sessions at a local community centre and twice-weekly exercise sessions at a private gym adjacent to the community centre. All participants were offered a referral for a consultation at a local sleep clinic.

During the first exercise session, participants underwent a one-on-one baseline assessment by a certified exercise physiologist or physiotherapist and were given an individualized exercise program to follow during the twice-weekly exercise sessions. Exercise sessions lasted for one hour and consisted of a personally tailored combination of aerobic, strength, and flexibility exercises. Participants were guided to adapt the intensity of the exercises based on their ability, which required attention to their own breath and body tension during the exercises. Individuals exercised in the same room as other members of their cohort.

The education component was developed based on evidence-based practice guidelines for FM and chronic pain self-management [10] and tested in a feasibility trial [16]. The 1-hour sessions took place weekly, immediately following one of the exercise sessions. Education included: four sessions focused on cognitive-behavioural strategies for improving mental health (facilitated by a doctoral student in clinical psychology and an occupational therapist, supervised by a registered psychologist), two sessions focused on reconceptualizing pain to improve exercise as well as integrating pain self-care techniques with therapeutic exercise and movement (facilitated by a physiotherapy pain specialist), two nutrition sessions (facilitated by a dietitian), and two peer support sessions (facilitated by a social worker). The total number of hours for the intervention was approximately 32.

Over the course of each 10-week intervention, a monthly healthcare provider “team huddle” teleconference sessions were conducted. These sessions provided a venue to ensure the integration of care and the opportunity to discuss issues or concerns regarding adverse events, patient care, and patient progress.

### Control group

The control groups were made up of ten individuals per cohort for a total of three cohorts running simultaneously with each intervention group. Participants randomized to the control group received usual care. In the catchment area for the current study, usual care for fibromyalgia involves a referral to a rheumatololgist. Therefore, patients assigned to the control group were referred to a local rheumatologist for a single, one hour, one-on-one consultation. During this appointment, the patient’s history was taken, a physical exam was performed, basic education about fibromyalgia was provided, and online resources for self-directed self-management were provided. At the conclusion of the study (i.e., three-month follow-up questionnaires were collected from all participants), the control group participants were offered the multidisciplinary group intervention.

### Measures overview

Sample characteristics including demographics (e.g., age, race/ethnicity, marital status) and clinical variables (e.g., time since diagnosis, comorbid conditions) were assessed during study enrollment. The primary and secondary outcomes were assessed prior to the intervention, immediately following the intervention, and at a three-month follow-up. The primary outcome was perceptions of quality of care and secondary outcomes were disease-related functioning, anxious and depressive symptoms, pain beliefs, and health service utilization. Consistent with guidelines secondary outcome measures were included as they lent evidence for the primary outcome [22].

### Primary outcome measure

The primary outcome, patient-perceived quality of care, was assessed by the 20-item Patient Assessment of Chronic Illness Care (PACIC) survey [20]. The measure has the following five subscales: patient activation and involvement, delivery system design and decision support, goal setting and tailoring, problem-solving and contextual counseling and follow-up care and coordination. The PACIC has demonstrated adequate reliability as well as face, construct, and concurrent validity [20]. In the current research, Cronbach’s alpha ranged from 0.95 to 0.97 across timepoints.

### Secondary outcome measures

The Fibromyalgia Impact Questionnaire (FIQ) is a 20-item patient report measure which evaluates different domains of FM [23]. Seven items measure patients’ ability to do work, pain, fatigue, rest, stiffness, anxiety, and depression. Two additional items measure the extent to which patients felt good and missed work. The FIQ has demonstrated reliability and responsiveness to change in clinical studies [23]. In the current research Cronbach’s alpha ranged from 0.85 to 0.88 across timepoints.

The Hospital Anxiety and Depression Scale (HADS) is a 14-item self-report measure of psychological distress which can be divided into two subscales reflecting depressive and anxious symptoms [24]. Psychometric properties of the HADS are adequate in primary care patients and the general population [25]. In the current research Cronbach’s alpha ranged from 0.88 to 0.93 across timepoints.

The brief version of the Survey of Pain Attitudes (SOPA-B) is 30-item patient report measure which assesses attitudes and beliefs about pain with adequate psychometric properties [26]. The SOPA-B assesses seven domains including pain control, disability, medical cure, solicitude, medication, emotion, and harm. In the current research, Cronbach’s alpha ranged from 0.70 to 0.79 across timepoints.

Health care utilization data was collected from the local health authority. Specifically, the number of emergency department visits participants made six months prior to the intervention, during the intervention, and for three months following the intervention were obtained. The ED data included all reasons for visiting the ED, whether FM-related or not.

### Statistical analysis

As a randomization check, demographic and clinical variables (e.g., age, gender, ethnicity, years since FM diagnosis) were compared between participants assigned to the control and intervention conditions using t-tests for continuous variables and Pearson’s chi-square or Fisher’s exact tests for categorical variables. Descriptive analyses were conducted on the demographic and clinical variables, as well as the primary and secondary outcome variables at each of the three timepoints. Patterns of missing data were analyzed using Little’s MCAR test. Following recently published guidelines no data was imputed and complete case analysis was conducted [27].

Regarding the main analyses, mixed model analyses using maximum likelihood estimation were used to assess group by time interactions. Independent sample t-tests were used to compare the intervention versus control group difference scores (baseline to post-intervention and baseline to three-month follow-up). The criteria for statistical significance was *p* < 0.05. Effect sizes were calculated, and can be interpreted as small (Cohen’s *d* ≥ 0.20), medium (Cohen’s *d* ≥ 0.50), and large (Cohen’s *d* ≥ 0.80) [28]. The preliminary descriptive and bivariate analyses were conducted using SPSS Version 27 (Armonk, NY: IBM Corp), and the main analyses were conducted using R Version 3.6.3 (Vienna, Austria: R Foundation for Statistical Computing).

## Results

### Sample characteristics

The final sample was predominantly comprised of Caucasian women with a mean age of 61.48 years who had a diagnosis of FM for an average of 14.64 years (range 1–57 years). Self-reported medical comorbidities were common and included headaches (n = 24; 40%), chronic fatigue syndrome (n = 21; 35%), high blood pressure (n = 17; 28.33%), and hypothyroidism (n = 15; 25%). In terms of severity of psychological distress using cut off scores ≥ 8 on the HADS at baseline, 38 (63.33%) participants had notable anxious symptoms scores and 30 (50%) participants had notable depressive symptoms. Baseline characteristics for the total sample and for the two study conditions are presented in Table 1.

**Table 1:** Baseline characteristics of overall sample and by treatment condition

	Overall sample (n = 60)	Intervention (n = 30)	Control Group (n = 30)	*p*
Age in years, mean (SD)	61.48 (11.07)	62.57 (10.54)	60.23 (11.75)	0.634
Female *n* (%)	56 (93.3%)	28 (93.3%)	28 (93.3%)	0.694
Race/Ethnicity				0.239
Caucasian *n* (%)	43 (71.7%)	23 (76.7%)	20 (66.7%)	
Indigenous *n* (%)	7 (11.7%)	1 (3.3%)	6 (20%)	
East or South East Asian *n* (%)	1 (1.7%)	1 (3.3%	0 (0%)	
Other *n* (%)	2 (3.3%)	0 (0%)	2 (6.7%)	
Information not available	7 (11.7%)	5 (16.7%)	2 (6.7%)	
Education level				0.126
Some high school *n* (%)	2 (3.33%)	1 (3.3%)	1 (3.3%)	
High school *n* (%)	4 (6.7%)	0 (0%)	4 (13.3%)	
Some college/university *n* (%)	11 (18.3%)	5 (16.7%)	6 (20%)	
College/university *n* (%)	39 (65%)	22 (73.3%)	17 (56.7%)	
Information not available	4 (6.7%)	2 (6.7%)	2 (6.7%)	
Household income				0.708
< $59,999	34 (61.8%)	18 (60%)	16 (53.3%)	
> $60,000	21 (38.2%)	10 (33.3%)	11 (36.7%)	
Information not available	5 (8.3%)	2 (6.67%)	3 (10%)	
Marital status				0.061
Single *n* (%)	7 (11.7%)	2 (6.7%)	5 (16.7%)	
Married/common-law *n* (%)	31 (51.7%)	14 (46.7%)	17 (56.7%)	
Separated/widowed *n* (%)	18 (30%)	12 (40%)	6 (20%)	
Information not available	4 (6.7%)	2 (6.7%)	2 (6.7%)	
Years since FM diagnosis, mean (SD, range)	14.64 (11.11, 1–57)	14.23 (13.02, 1–57)	15.05 (9.11, 1–30)	0.510

### Preliminary analyses

Details regarding participant flow through the intervention are presented in Fig. 1. The majority of intervention group participants completed the intervention and post-treatment study questionnaires (n = 28; 93.33%), and almost two-thirds completed the three-month follow-up questionnaires (n = 19; 63.33%). Additionally the majority of the control group participants completed the post-treatment study questionnaires (n = 22; 73.33%) and the three-month follow-up questionnaires (n = 19; 63.33%).

Descriptive statistics for the primary and secondary outcome measures at pre-intervention, post-intervention, and three-month follow-up are presented in Table 2. Random assignment to condition was successful, with only one difference emerging between the intervention and control groups at baseline. Specifically, the control group reported higher scores on the control subscale of the SOPA-B at baseline (M = 2.46, SD = 0.70) compared to the intervention group [M = 2.08, SD = 0.72), *t*(57) = 2.05, *p* = 0.045]. No other baseline variables were significantly different between the intervention and control groups (all *p*’s ≥ 0.116).

**Table 2:** Means and standard deviations of study variables

Variable	Intervention group			Control group		
	Baseline mean (SD)	Post mean (SD)	3-month mean (SD)	Baseline mean (SD)	Post mean (SD)	3-month mean (SD)
Primary outcome						
PACIC						
Patient activation	2.41 (1.19)	2.60 (1.23)	2.36 (1.41)	2.47 (1.31)	2.20 (1.07)	2.52 (1.22)
Decision support	2.31 (1.07)	2.58 (1.34)	2.26 (1.29)	2.30 (1.15)	1.93 (1.06)	2.50 (1.45)
Goal setting	1.83 (.90)	2.34 (1.31)	1.91 (1.19)	1.93 (1.11)	1.81 (1.00)	1.90 (1.07)
Problem solving	2.16 (1.02)	2.39 (1.29)	2.21 (1.42)	2.51 (1.45)	1.90 (1.05)	1.99 (1.17)
Follow-up	1.73 (0.74)	2.15 (1.00)	1.78 (1.07)	1.93 (1.07)	1.62 (0.98)	1.84 (0.99)
Secondary Outcomes						
FIQ						
Physical impairment	3.76 (2.43)	3.44 (2.30)	3.35 (2.36)	4.73 (2.22)	5.19 (2.26)	5.01 (1.93)
Feel good	10.11 (2.83)	8.63 (3.35)	7.94 (3.13)	9.93 (3.16)	8.94 (3.11)	8.73 (2.90)
Work missed	4.55 (3.46)	2.56 (2.49)	2.78 (3.55)	4.95 (3.58)	4.77 (2.94)	4.85 (3.37)
Do job	6.97 (1.92)	5.71 (2.65)	6.00 (2.71)	6.57 (2.27)	6.89 (2.23)	6.33 (1.88)
Pain	7.10 (1.90)	5.93 (1.94)	6.23 (2.17)	6.83 (1.51)	6.90 (1.92)	6.11 (1.94)
Fatigue	7.67 (2.32)	7.07 (2.50)	6.88 (2.48)	8.31 (1.26)	7.70 (1.95)	7.44 (2.06)
Rest	7.10 (2.68)	6.00 (2.77)	6.10 (2.57)	7.41 (2.46)	6.90 (2.27)	6.06 (3.06)
Stiffness	7.43 (1.72)	6.50 (2.34)	6.55 (2.63)	7.48 (1.72)	7.45 (2.09)	7.12 (2.18)
Anxiety	5.57 (3.07)	4.74 (3.43)	4.65 (3.38)	5.64 (2.63)	4.40 (2.52)	4.17 (3.01)
Depression	5.00 (3.39)	4.19 (2.69)	3.80 (3.00)	4.44 (2.93)	4.90 (2.97)	4.50 (3.45)
Total	53.29 (14.43)	46.85 (16.00)	45.48 (15.51)	54.13 (11.68)	52.38 (12.34)	49.13 (14.63)
HADS						
Anxiety	9.27 (4.35)	8.00 (5.34)	8.75 (4.66)	8.85 (4.77)	7.21 (3.82)	8.90 (5.04)
Depression	7.77 (3.94)	6.04 (3.47)	6.25 (4.25)	7.43 (4.40)	7.32 (4.82)	8.60 (5.11)
SOPA						
Control	2.08 (0.72)	2.50 (0.67)	2.71 (0.53)	2.46 (0.71)	2.26 (0.68)	2.56 (0.40)
Emotion	2.59 (1.19)	2.78 (1.02)	2.67 (1.00)	2.76 (0.75)	2.81 (0.57)	2.82 (0.77)
Disability	2.73 (0.62)	2.33 (0.94)	2.45 (1.03)	2.46 (1.03)	2.80 (1.08)	2.94 (0.89)
Harm	1.21 (0.80)	0.86 (0.61)	1.32 (0.78)	1.48 (0.88)	1.53 (0.90)	1.24 (0.52)
Medication	2.01 (1.17)	1.95 (0.99)	1.96 (1.05)	2.18 (0.92)	2.37 (1.00)	2.62 (0.96)
Solicitude	1.28 (1.01)	1.13 (0.88)	1.56 (0.95)	1.40 (0.89)	1.46 (0.75)	1.80 (0.90)
Medical cure	1.64 (0.98)	1.43 (0.83)	1.46 (0.74)	1.60 (0.92)	1.65 (0.71)	1.51 (0.68)

*PACIC* Patient Assessment of Chronic Illness Care survey, *FIQ* Fibromyalgia Impact Questionnaire, *HADS* Hospital Anxiety and Depression Scale, *SOPA* brief version of the Survey of Pain Attitudes

### Main analyses

#### Primary outcome

Results of the independent t-tests comparing difference scores for the intervention and control groups are presented in Table 3. Statistically significant, medium effect sizes were found post-intervention for the goal setting (Cohen’s *d* = 0.66) and follow-up care (Cohen’s *d* = 0.61) subscales of the PACIC. Notable effect sizes were also found for the problem solving (Cohen’s *d* = 0.55) and decision support (Cohen’s *d* = 0.47) subscales, but significance levels were *p* > 0.05. At the 3-month follow-up, change scores were not statistically different between groups and effect sizes were negligible to small, Cohen’s *d* ranging from 0.13 to 0.27.

**Table 3:** Difference score results for study variables

Variable	Difference Score (pre vs post)		Difference score (pre vs 3 month post)	
	Cohen’s d	*p*	Cohen’s d	*p*
PACIC				
Patient activation	0.23	0.27	0.21	0.29
Decision support	0.47	0.09	0.13	0.39
Goal setting	0.66	0.04	0.24	0.29
Problem solving	0.55	0.11	0.27	0.25
Follow-up	0.61	0.03	0.13	0.37
FIQ				
Physical impairment	0.45	0.07	0.42	0.11
Feel good	0.06	0.43	0.10	0.39
Work missed	0.77	0.02	0.38	0.17
Do job	0.69	0.02	0.36	0.15
Pain	0.59	0.03	0.03	0.46
Fatigue	0.07	0.42	0.08	0.41
Rest	0.30	0.18	0.36	0.15
Stiffness	0.45	0.08	0.11	0.37
Anxiety	0.11	0.36	0.38	0.15
Depression	0.91	0.01	0.73	0.045
Total	0.70	0.02	0.19	0.30
HADS				
Anxiety	0.12	0.35	1.08	0.00
Depression	0.87	0.02	0.12	0.38
SOPA				
Control	0.82	0.01	0.72	0.03
Emotion	0.20	0.26	0.17	0.32
Disability	0.67	0.03	0.38	0.14
Harm	0.61	0.03	0.22	0.26
Medication	0.30	0.46	0.22	0.26
Solicitude	0.27	0.19	0.15	0.33
Medical cure	0.49	0.06	0.56	0.06

#### Secondary outcomes

T-test results for the secondary outcomes are presented in Table 3. Significant differences (*p* < 0.5) were found post-intervention for FIQ depression (Cohen’s *d* = 0.91), FIQ ability to do job (Cohen’s *d* = 0.69), FIQ pain (Cohen’s *d* = 0.59), FIQ work missed (Cohen’s *d* = 0.77), FIQ total (Cohen’s *d* = 0.70), and HADS depression (Cohen’s *d* = 0.87). Except for FIQ depression (Cohen’s *d* = 0.73), these effects were no longer significant at the three-month follow-up. Of note, at the three-month follow-up there was a significant improvement in HADS anxiety (Cohen’s *d* = 1.08).

Group t-tests of the difference from baseline to post-intervention also revealed significant differences (*p* < 0.5) for the following SOPA-B subscales: control (Cohen’s *d* = 0.82), disability (Cohen’s *d* = 0.67), and harm (Cohen’s *d* = 0.61). Except for control (Cohen’s *d* = 0.72) all other effects were no longer significant at the three-month follow-up.

With respect to health care utilization, no differences were detected between baseline data (ED visits over the six months leading up to the intervention) compared to access during the intervention (ED visits over three months encompassing the intervention), or for the three months following the intervention. When analyses were conducted specifically for pain-related visits to the ED, as well as for ED visits for all reasons, the findings remained the same with no significant differences between treatment and control conditions.

#### Adverse events

The potential for adverse events were monitored throughout the trial, and was discussed during the monthly team meetings. No adverse events occurred.

## Discussion

This pilot research examined the effectiveness of a community group-based multidisciplinary program as compared to usual care for individuals diagnosed with FM. At the completion of the 10-week intervention, small to medium size effects were found on the primary outcome, patient assessment of chronic illness care. At the conclusion of the intervention, small to medium sized significant effects of the intervention were also found on a number of the secondary outcomes. Participants reported lower levels of pain, an increased ability to do work, including housework, and missing less work throughout the week. Additionally, participants reported decreased depressive symptoms, increased adaptive beliefs about pain, and decreased maladaptive beliefs about pain.

Patient-reported outcome measures include direct reports by the patient of their healthcare outcomes associated with either illness or treatment [29]. In the current research, the PACIC was used to assess participants’ satisfaction with treatment. At the conclusion of the intervention, participants reported small to medium improvements in goal setting and follow-up care. These results suggest that the intervention improved quality of care from the patient’s perspective, although effects were lost at the three-month follow-up timepoint.

To assess the efficacy of interventions for FM clinical trials the use of the FIQ has been recommended [30]. Following the intervention, significant improvements were found across multiple subscales of the FIQ, including the total FIQ score, suggesting that the intervention decreased the functional impact of FM on participants. Additionally, participants reported an increased ability to do work, decreased pain, and missing less work suggesting that they had the capacity to engage more fully in their day-to-day lives.

A large proportion of participants (50%) had depressive symptoms at the beginning of the intervention, and these depressive symptoms were significantly reduced following the intervention as assessed by both the FIQ and the HADS, with a medium effect size remaining for FIQ depression at the three-month follow-up. No effects were found for the depression subscale of HADS at the three-month follow-up. One potential explanation for this could be that HADS items were developed specifically to exclude physical and cognitive symptoms, and instead focusing on emotional aspects of depression, whereas FIQ depression was assessed with a single item that could be interpreted by respondents more broadly. Of note, anxiety as assessed by the HADS was significantly reduced at the three-month follow-up. This could be related to improvements in patient perceptions of the quality of their health care, as well as more adaptive pain beliefs.

One of the most marked findings of the current research was increased adaptive pain beliefs among patients in the intervention group, compared to usual care. Psychological factors play an important role in the experience of persistent pain [31]. In particular, a lack of controllability over pain has been linked to increased suffering [32]. At the conclusion of the intervention, improvements were found in the belief in the ability to control one’s pain, the extent to which one believes their pain is disabling, the belief that pain will lead to physical damage, and that exercise should be avoided. These findings could be linked to elements of this program including a tailored exercise plan and supported twice weekly exercise as well as the focus on self-management skills. These results are consistent with other research studies which have measured changes in an individual’s ability to cope with their illness following participation in a psychoeducational program [33]. The incorporation of cognitive behavioural therapy in particular has been shown to be beneficial and has resulted in improvements in the ability to cope with FM [34].

With the exception of depression assessed by the FIQ, anxiety assessed by the HADS, and beliefs about the controllability of pain assessed by SOPA-B, significant improvements observed among patients in the treatment condition were lost at the three-month follow-up. One potential explanation could be the relatively small number of study participants that returned the questionnaires at three months post-intervention, resulting in a loss of statistical power to detect significant effects. Another potential explanation could be the lack of support for patients following the end of the intervention as there was no ongoing organized support. Given that there is no standardized treatment of FM, integral to the success of supported self-management is an ongoing collaborative relationship between the patient and their health care providers [35, 36]. Future research may consider the benefits of working with healthcare professionals to put together a personalized plan for long-term maintenance, including close family members in this planning [37], and coordinating ongoing care with accessible community-based resources including peer supports [38, 39].

A review of FM management guidelines found that organizations have assigned the highest rank of treatment recommendations to multicomponent therapies as well as aerobic exercise and cognitive behavioral therapy [40]. Indeed, Canadian recommendations identify the ideal care of FM as incorporating a multimodal approach with active participation of the FM patient [10]. The findings of this intervention supports the potential value of community-based multidisciplinary interventions for FM, and indeed, participation in community-based programs has been recommended due to its accessibility and affordability [1]. In terms of implications for practice, the ease with which this community-based multidisciplinary group intervention can be executed speaks to its sustainability, as well as its ability to be implemented outside a major urban centre. Specifically, involvement of key health professionals (e.g., physiotherapists, occupational health therapists, and pain management specialists) is required. Support from health authority stakeholders is also critical and providing occasional education for this type of intervention could be written into the job descriptions of these care providers. An implemented program such as this could become an immediate first step following an initial diagnosis with FM. This could help improve health services access and help to more quickly transition individuals on a path to improved function with an array of self-management tools. Further, given the findings of this research, it may be beneficial to extend this type of model to give access to multidisciplinary care to smaller communities via remote technologies, including educational sessions and guided exercise.

Several limitations of the current research should be acknowledged. First, the sample was comprised mainly of middle-aged Caucasian women and therefore the generalizability of the findings to other genders, ages, and ethnicities may be limited. The second limitation relates to the relatively small sample size. Our power analysis indicated the sample size collected would be large enough for the primary outcome measures, but a larger sample might have allowed the detection of more significant changes across outcomes and at the follow-up assessment. An additional limitation is the healthcare utilization data in the current study focused on emergency department visits. Future research would benefit from incorporating more information from a variety of sources including visits to general practictioners. Finally, some of the treatment sessions were delivered by different therapists across the three cohorts and therapist-related factors could not be controlled for in the analyses.

In conclusion, FM is a pervasive and complex clinical syndrome. The findings of the current research indicate that multi-disciplinary, community-embedded group treatment of FM was efficacious at improving patient perceptions of quality of care, reducing pain severity, reducing depressive symptoms, and improving the amount of work, including housework, that participants were previously incapable of performing. Notably the intervention had a particularly powerful impact on patients’ perceptions that they can control the pain that they experience.

## Data Availability

The participants of this study did not give written consent for their data to be shared publicly, so due to the sensitive nature of the research supporting data is not available.
